# MicroRNAs in Valvular Heart Diseases: Biological Regulators, Prognostic Markers and Therapeutical Targets

**DOI:** 10.3390/ijms222212132

**Published:** 2021-11-09

**Authors:** Francesco Nappi, Adelaide Iervolino, Sanjeet Singh Avtaar Singh, Massimo Chello

**Affiliations:** 1 Department of Cardiac Surgery, Centre Cardiologique du Nord de Saint-Denis, 93200 Paris, France; 2 Department of Cardiovascular Sciences, Fondazione Policlinico Universitario A. Gemelli IRCSS, 00168 Rome, Italy; adelaide.iervolino@libero.it; 3 Department of Cardiothoracic Surgery, Golden Jubilee National Hospital, Glasgow G81 4DY, UK; sanjeetsingh@nhs.ne; 4 Cardiovascular Surgery, University Campus Bio-Medico di Roma, 00128 Rome, Italy; M.Chello@unicampus.it

**Keywords:** miRNAs, valvular heart diseases, aortic stenosis, calcification, mitral valve prolapse, aortic valve defect, vectors, delivery systems, nanoparticles

## Abstract

miRNAs have recently attracted investigators’ interest as regulators of valvular diseases pathogenesis, diagnostic biomarkers, and therapeutical targets. Evidence from in-vivo and in-vitro studies demonstrated stimulatory or inhibitory roles in mitral valve prolapse development, aortic leaflet fusion, and calcification pathways, specifically osteoblastic differentiation and transcription factors modulation. Tissue expression assessment and comparison between physiological and pathological phenotypes of different disease entities, including mitral valve prolapse and mitral chordae tendineae rupture, emerged as the best strategies to address miRNAs over or under-representation and thus, their impact on pathogeneses. In this review, we discuss the fundamental intra- and intercellular signals regulated by miRNAs leading to defects in mitral and aortic valves, congenital heart diseases, and the possible therapeutic strategies targeting them. These miRNAs inhibitors are comprised of antisense oligonucleotides and sponge vectors. The miRNA mimics, miRNA expression vectors, and small molecules are instead possible practical strategies to increase specific miRNA activity. Advantages and technical limitations of these new drugs, including instability and complex pharmacokinetics, are also presented. Novel delivery strategies, such as nanoparticles and liposomes, are described to improve knowledge on future personalized treatment directions.

## 1. miRNAs Effects on Aortic Stenosis and Mitral Prolapse Necessity of Novel Biomarkers

### 1.1. miRNAs Involvement in Aortic Stenosis Pathogenesis and Calcification Progression

microRNAs (miRNAs) belong to non-coding RNAs and are generally transcribed from DNA into primary miRNAs and subsequently processed into precursor miRNAs and mature miRNAs [1]. They usually possess translational regulatory functions, exerted by interaction with 3′ untranslated region (3′ UTR) of target miRNAs [1]. Aberrant miRNA levels have been demonstrated in multiple diseases and, therefore, constitute useful markers of prognostic importance [2,3,4]. Recently, Carthew et al. investigated the role of two leading classes of these small RNAs with specific regulatory functions and found direct implications in biological processes as well as in the etiology and treatment of diseases [5]. Authors identified short interfering RNAs (siRNAs) and miRNAs’ pivotal role in eukaryotic, somatic, and germ lines. The specific targets of siRNAs and miRNAs were discovered to be the genome sentinels through the ability to negatively regulate gene expression in the post-transcriptional phase [5].

In aortic stenosis, miRNAs were also found to be altered in stenotic aortic valve leaflets compared to insufficient ones by Nigam et al. [6]. The reduced levels of miR-26a, miR-30b, and miR-195 reported in stenotic valves, were almost certainly responsible for their higher risk of developing valvular leaflet fusion. The change of morphology is due to accelerated calcium accumulation compared to regurgitant aortic valves without morphological fusion of valve leaflets. These miRNAs were involved in the biological processes that modulate calcification-related genes in vitro [6]. Other studies have confirmed miRNAs’ involvement in calcification pathways and will be discussed later.

Several studies have then recorded an important association of miRNAs with post-transcriptional regulation of gene expression in aortic valve stenosis. miR-141 was found to be a regulator of the levels of bone morphogenetic protein 2 (BMP-2), whereby unrestrained activity led to calcification of the aortic valve mediated by a stimulation of osteogenesis. miR-141 was markedly attenuated in patients with aortic stenosis associated with the bicuspid aortic valve [7]. Yanagawa et al. proposed this new key role of miR-141 in the modulation of aortic valve calcification disorders, highlighting the strategic therapeutic target that emerged in the assessment of progressive calcification in stenotic aortic valve disease [6,7]. The peculiar morphologic features of the stenotic aortic valve may probably be explained by the lower expression of miR-30b which is a known repressor of bone morphogenetic protein 2-mediated osteogenesis [7].

**Figure 1:**
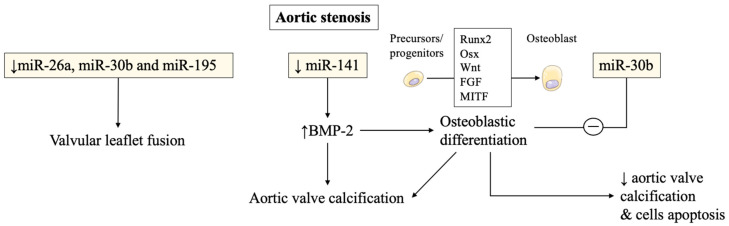
The role of miRNAs in aortic stenosis severity and valve calcification. Reduced levels of miR-26a, miR-30b, and miR-195 contribute to further damage to the valve. The negative regulation exerted on osteoblastic differentiation by miR-30b favors a better prognosis, in terms of a decrease in valve calcification. On the other hand, reduced levels of miRna-141 guarantee osteoblastic calcification processes via increasing BMP-2 levels, a relevant factor involved in bone production. Osteoblasts differentiate from precursors and progenitors through several factors, including Runx2 and Wnt. This differentiative process negatively affects valves elasticity by contributing to their calcification. BMP: bone morphogenetic protein. Runx2: RUNX Family Transcription Factor 2, OSX: osterix factor, FGF: Fibroblast growth factor, MITF: Microphthalmia-associated transcription factor.

Zhang et al. demonstrated the role of miR-30b in reducing osteoblast differentiation activity induced by bone morphogenetic protein 2 [8]. The latter was implicated in promoting calcific aortic valve disease. The expression of miR-30b was found effective in reducing the risk of human aortic valve calcification and apoptosis through direct targeting of Runx2, Smad1, and caspase-3 [8] (Figure 1).

Song et al. analysed the progression of calcified aortic valve disease and suggested the crucial role of myofibroblastic and osteoblast-like phenotypes [9]. The authors reported substantial differences in the levels of two miRNA classes: miR-486 and miR-204. The levels of miR-486 were increased in calcific aortic valves where myofibroblastic transition was induced by upregulation of the expression of Runx2 and Osx and miR-204 levels were deficient, which lead to high cellular and valvular pro-osteogenic activity. This study demonstrated a sophisticated modulation of the epigenetic mechanisms that support pro-osteogenic pathways, paving the way for their therapeutic potential [9]. The differential expression of miRNAs in the bicuspid aortic valve has been proved, with evident calcific-degenerative changes differentiating the bicuspid morphology from the tricuspid one [9].

### 1.2. The Importance of Discovering Diagnostic Biomarkers for Mitral Valve Prolapse

Plasma levels of miRNAs have been used to monitor degenerative disease of the mitral valve (MV) but have not been widely adopted by the cardiology community [4,6,7,9,10,11]. The lack of enthusiasm of cardiologists for investigations into degenerative aortic valve stenosis [6,7,8,12,13] can be partially deduced from the lack of positive findings in mitral valve degenerative diseases leading to regurgitation [14,15,16,17,18,19].

Mitral valve prolapse is a debilitating disease with a worldwide prevalence of 2–3% [20,21]. In younger patients, pathoanatomic features arise because of excessive tissue formation on mitral valve leaflets leading to Barlow’s syndrome [22] while pathoanatomic lesions in older patients tend to manifest as fibroelastic degeneration. Both forms of the disease can lead to leaflet prolapse and chordal elongation or rupture [23,24,25].

Mitral valve prolapse severity can be assessed by transthoracic echocardiography. Although the information provided by 2D (bi-dimensional) and 3D (three-dimensional) echography yields a comprehensive assessment [26,27,28], the lack of effective medical therapy makes surgery the only viable treatment option [29,30,31]. Recently, new techniques for mitral valve repair have been developed by transcatheter approaches [32,33,34].

The landmark study by Mayeux et al. has highlighted the emergence of biomarkers as an important tool but there is still no consensus for using specific circulating biomarkers for mitral valve prolapse diagnosis in clinical practice [4].

The degenerative process involving the mitral valve has been addressed by several investigators looking for circumstantial associations between the presence of biomarkers and the histopathological alteration. Songia et al. suggested the possible association between osteoprotegerin and mitral valve degeneration in Barlow syndrome without recording a specific correlation [14,15]. Tan et al. used the proteomics method and evaluated the presence of selective biomarkers, thus directing the investigation in the same field [16]. Although the investigators have recorded higher levels of haptoglobin, platelet basic protein, and complement component C4b in individuals with degenerative mitral valve prolapse, the evidence was not conclusive for a specific correlation between biomarkers and disease development [16].

## 2. Pathophysiology of Valvular Calcification Pathways, from Preclinical Models to Clinical Perspectives

### 2.1. Complex Interplays between miRNAs and Intracellular Osteogenic Signals

Calcium phosphate crystals are responsible for abnormal accumulation in either native or prosthetic valves, leading to valvular calcification (VC), loss of elasticity, and ischemic conditions [35]. Intimal and medial layers of major vessels can display calcifications, the former being associated with atherosclerotic phenotypes, the latter with common cardiovascular risk factors including diabetes and osteoporosis. Some authors propose that vascular smooth muscle cells (VSMCs) begin the process by undergoing phenotypical changes to an osteoblastic nature and losing contractile markers such as smooth muscle 22 alpha (SM22α) and alpha-smooth muscle actin (α-SMA) [36].

Several coding genes have been studied and linked to the specific development of calcific valves. Osteopontin (OPN), osteocalcin (OC), bone morphogenetic proteins (BMPs), alkaline phosphatase (Alp), and transcription factor Runx2 were demonstrated to be upregulated in calcific processes [35]. Other described signalling pathways include exosomes crosslinking among the three layers of the vascular wall and Wnt/Beta-catenin, and advanced glycation end products (AGEs) [37,38]. Osteoprotegerin/receptor activator of nuclear factor-kB and its ligand (OPG/RANK/RANKL) have also been noted to interact between either the intima and media layers or the media and adventitial layers [37,38].

Pro-inflammatory activity has also been related to VC. Tumor necrosis factor-alpha (TNFa), interleukins including IL-1B and IL-6, tumor growth factor-beta 1 (TGFB1), and other cytokines mediate vascular smooth muscle cells transition into osteoblast-like cells [39]. The phenomenon is known to be enhanced by reactive oxygen species (ROS) production and determined by the discovery of cytokines and factors expression in the aortic tissue. A Canadian preclinical study by Agharazii et al. highlighted the valvular calcification processes developing from chronic kidney disease, were increased in rats by cytokines [39]. Interleukin-1β, interleukin-6, and tumor necrosis factor were overexpressed in aortic tissues. Additionally, nicotinamide adenine dinucleotide phosphate (NADPH) oxidase expression was increased while antioxidant enzymes (SOD1, SOD2, Gpx1, and Prdx1) demonstrated lower levels than normal [39].

MicroRNAs, with the exception of miR-29, have been categorized into either activating or promoting valvular calcification. Some authors claim miR-29a/b repression is a pivotal factor in calcification generation. A preclinical study by Du et al. revealed how increased expression of ADAMTS-7 (a disintegrin and metalloproteinase with thrombospondin motifs-7), caused marked upregulation of calcifying rat vascular smooth muscle cells, and was linked to miR-29 inhibition [40]. Calcification inhibitors miRNAs, which roles were revealed by decreasing expression in calcific tissues, have been described by in vitro and in vivo studies, in both animals and humans. MiR-30b, 30c were found to directly inhibit Runx2 factors, such as miR-133a, miR-204, and miR-205 [41,42,43,44].

Recently, Lin et al. demonstrated miR-34c/5p to be downregulated in calcific tissues (in vitro high glucose-induced human aorta VSMCs) and inhibiting BMF as the primary target [45]. In fact, miR-34c from the miR-34 family is reported to participate in osteoblast differentiation [46,47]. It targets SATB2, a nuclear matrix protein that inhibits the expression of HOXA2, in turn negatively regulating Runx2 and increasing the activity of Runx2 and activator transcription factor 4 (ATF4). Runx2 and ATF4 are sophisticated regulators of osteoblastic differentiation, from osteochondral progenitors. Osterix is another important involved factor [48]. Investigators also claim miR-34c regulates Osteocalcin and other genes whose expression is controlled by Runx2 and SATB2 or ATF4 and SATB2 [48].

### 2.2. Over and Underexpression of miRNAs from In Vivo Animal Experimentations

Recently, investigators have provided proof of upregulated levels of miRNAs in calcific vessels. An in vitro demonstration of human and murine aortic tissue and, specifically, of smooth muscle cells expressing miR-29b at increased levels comes from a Japanese investigation [49] and a Spanish preclinical study [50].

Calcium deposition in human VSMCs favored by phosphorus (Ph) was evaluated by Sudo et al. to determine the impact of miR-29. Real-time quantitative PCR (RT-qPCR) analysis on Pi-induced calcific VSMCs was performed and showed decreased levels of elastin with consequent osteoblast-related genes expression. Of note, miR-29 was found to elicit elastin suppression, thereby closing the circle [49].

Panizo et al. induced vessel calcification in rats by using the common experimental method of feeding them a high phosphate diet. They found low levels of miR-133b and miR-211 alongside high levels of miR-29b [50]. The former correlated with overexpression of osteogenic RUNX2, the previously described factor, while the latter with lower expression of inhibitors of osteoblastic differentiation. The reliability of the study is conferred by in-vitro affirmation of the results: authors analyzed miR-29b, miR-133b, and miR-211 to demonstrate how they regulated the calcification process [50]. MiR-29-mediated elastin down-regulation also promotes osteoblastic differentiation [49].

To further evaluate the role of miRNAs in valvular calcification, exosomes from VSMCs have been evaluated by several authors through RT-qPCR. Interestingly, preclinical studies show differences in the expression of hundreds of miRNAs when comparing mice calcific models versus the normal population [51,52].

Pan et al. established a cellular calcification model using the mouse line MOVAS-1 [51]. To search for calcification, Alizarin Red staining was performed and differential miRNAs profiles were sequenced. Results showed 987 miRNAs to be upregulated in the cellular calcification model and 92 to be down-regulated, even though not all of them were showing significant *p*-values of comparison between the two populations’ expression [51].

### 2.3. Diagnostic and Prognostic Relevance of miRNAs in Mitral Valve Diseases

Over the recent years, miRNAs have represented an emerging class of widely labored circulating biomarkers in various pathological states including degenerative mitral valve disease [14,15,16].

Concerning the prolapse of the mitral valve, only a small number of circulating miRNAs have been studied focusing on the degenerative disorders of the MV. These findings were limited to the experimental animal models. Hulanicka et al. evaluated plasma miRNA levels as potential biomarkers of chronic myxomatous mitral disease (MMVD) in dachshunds. Investigators evaluated the expression of 9 miRNAs that had already been discovered and were involved in cardiovascular diseases [17]. Using a real-time PCR method, they found that the plasma levels of two out of nine miRNAs were significantly downregulated, recording an evident involvement of these molecules in developing endocardiosis in dogs [17].

In greater detail, according to the American College of Veterinary Internal Medicine (ACVIM), the plasma levels of miRNAs were compared in three groups of dogs. The authors recorded that miR-30b expression differed between dogs of the ACVIM group in stage B (asymptomatic n = 8) and those that were included in the unaffected stage A group (control N = 8) [17]. The expression of miR-133b was unlike in the ACVIM stage C group, in which mild to moderate heart failure occurred, as compared to that of stage A group of dogs. 9 miRNAs (including miR-125, miR-126, miR-21, miR-29b, and miR-30b) showed a downregulation trend only in the ACVIM stage C group recording non-significance for the expression of these classes of miRNA [17]. The levels of miR-423 were similar between healthy and sick dogs. The expression of miR-208a and 208b was not detected, suggesting they were completely downregulated. The plasma level of miR-30b could be correlated as a potential biomarker of ACVIM stage B heart failure in Dachshunds who developed endocardiosis while miR-133b expression could be correlated as a potential biomarker of ACVIM stage C. It should be noted that the lack of expression or notable change in expression in 7 miRNAs which were potential biomarkers for the development of heart disease in humans highlight the lack of transferability from animal models to clinical applicability [17].

A second study reported the miRNA expression profile in dogs suffering from MMVD (myxomatous mitral valve disease [18]). Li et al. quantified 277 miRNAs using RT-qPCR and compared three groups of 6 dogs [18]. The first group enrolled asymptomatic animals with no disease (ACVIM stage A control group). The second group included dogs exhibiting MMVD with mild to moderate enlargement of the heart chambers (ACVIM Stage B1/B2). The third group included animals with MMVD and congestive heart failure (ACVIM Phase C/D). For eleven miRNAs, the study results showed a different expression with a False Discovery Rate < 0.05. Dogs enrolled in group B who had stage B1/B2 disease or those included in group C who had stage C/D myxomatous mitral valve disease (MMVD) recorded four upregulated miRNAs. These two groups included three cfa-let-7/cfa-miR-98 family members with upregulation, while seven others were downregulated when compared to the stage A control group. The evidence suggested significant differences in the expression of six of the 11 miRNAs when comparing animals belonging to stage C/D and those that were included in stage B1/B2. Furthermore, the most marked changes in miRNA expression were associated with an increase in the severity of MMVD. The significance of this study relates to the fact that these miRNAs can be candidates biomarkers, providing further insights into specific gene regulation pathways in MMVD that developed in dogs [18] (Table 1).

The major concern with the use of animal models to evaluate the expression of miRNA is the increased risk of discrepancy with human pathoanatomic conditions. Bulent et al. worked around this problem by investigating the expression profile of circulating miRNAs in the development of mitral chordae tendineae rupture in humans [19]. 22 miRNAs were studied in patients who developed mitral regurgitation due to progressive degeneration of the connective structure of the chordae tendineae leading to rupture. Evidence has suggested that the downregulation of various miRNA classes in patients with mitral valve degenerative disease led to rupture of chordae tendineae [19]. Using bioinformatics analysis, the authors indicated the following target genes involved in MCTR (MMPs, TIMP-2, TGFBR2, VEGFA, PIK3R2, NRAS, PPP3CA, PPP3R1, PTGS 2) which were regulated by 13 miRNAs [19].

Songia et al. performed the first study using human plasma from patients with degenerative mitral valve prolapse and noted a strong correlation between several circulating miRNAs and mitral valves with myxomatous prolapse [53]. Some of the tested miRNAs were also overlapping with those from Bulent et al. [19] and a similarity emerged: lower levels of miRNA 223-3p were found in both patients suffering from the prolapse of the MV and patients who developed MCTR [53] (Table 2).

The authors working on circulating biomarkers have provided valuable information on the etiology of degeneration and prolapse of MV alongside the possibility of stratifying patients affected by the disease [53]. Deroyer et al. investigated the role of apolipoprotein-A1 in fibroelastic disorders of MV revealing that the biomarker was indicated as an independent predictor of MR gravity [54].

In another study, Tan et al. performed an analysis using proteomic evaluation on two pooled plasma samples from 24 individuals affected by mitral valve prolapse and MR compared to 24 individuals with no MV prolapse and failure [16]. All enrolled patients received combinatorial peptide ligand library (CPLL) beads prior to iTRAQ labeling and ESI-MS/MS. The investigators noted a decrease in circulating levels of plasma haptoglobin, basic platelet protein, and complement component C4b in patients who developed MR due to fibroelastic deficiency compared to those without degenerative MV disorders. The results were confirmed with the ELISA test which was performed in all 48 patients enrolled in the study and matched 48 additional individual ELISA tests [16].

Unlike the studies cited above, the results reported in the analysis by Songia et al. were supported by solid statistical evidence, underlined by a marked change in plasma level of the miRNAs (miR-150-5p, miR-451a, and miR-487a-3p) studied using AUROC curves [53]. The keynote of the study was the fact that the authors assessed a cell-type enrichment analysis, based on validated miRNAs, revealing the existence of specific cell populations morphogenetically linked to different cardiovascular tissues, including the morphogenetic specificity of the mitral valve tissue. This suggested a link between ERBB and JAK-STAT signaling pathways as potentially relevant to understanding the recently discovered mechanisms involved in the evolution of mitral valve prolapse [53].

Among other things, the study of Songia et al. confirmed the existence of well-characterized signaling pathways involved in cell migration and proliferation of endothelial cells [15,16,55,56] as well as in the deregulation of homeostasis of the extracellular matrix [57,58,59]. In particular, the authors demonstrated that patients who develop degenerative disease of the mitral valve, either in the fibroelastic or myxomatous form, recorded a different expression of miR-150-5p leading to several pathological processes including fibrosis and neoplastic proliferation [60,61,62].

Although the report of Songia et al. claimed that specific circulating biomarkers could be interpreted as molecular signatures, the study has some critical points [53]. The study lacks numerically validated data in the different miRNA expressions between patients who had fibroelastic degenerative disease of MV and those who instead presented a myxomatous degeneration typically related to Barlow syndrome. In addition, the cohort of patients studied concerns those with degenerative prolapse of the MV with severe MR and eligible for surgical treatment. In fact, the study did not report any evaluation of the plasma levels of miRNAs capable of identifying a larger cohort of individuals with a prolapse of the MV coexisting with a mild or moderate mitral insufficiency [53].

However, given the reported evidence, the future direction postulates that miRNAs identified in plasma could be used soon and is an inexpensive screening tool for patients with progressive degenerative mitral valve disease and severe mitral regurgitation.

## 3. Altered miRNAs Expression in Congenital Valve Disorders and Cardiogenetic Processes

Congenital heart diseases (CHD) comprise a large group of functional and structural disorders, namely atrial septal defects (ASD), ventricular septal defects (VSD), pulmonary valve atresia (PVA), coarctation of the aorta (CoA), tricuspid atresia (TA), tetralogy of Fallot (TOF) and several others [63,64,65]. miRNAs play a pivotal role in heart development. The process of cardiac tissue formation and expression requires precise regulation and single miRNAs studies have been addressed in past decades. miR-1 and miR-133 are transcribed in a tissue-specific manner during development [66]. MiR-1 targets HDAC4, histone deacetylase 4 which is a repressor for muscle gene expression, thereby stimulating myogenesis. miR-133 inhibits serum response factor (SRF) and promotes the differentiating process [66].

Several other preclinical studies, conducted on zebrafish ventricles, denoted the role of miR-133 to diminish cardiac regenerating processes [67]. Following resection of zebrafish ventricular apex, a reduced expression of miR-133, coupled with increased regenerative potential, has led to this concept [67].

Cardiogenesis is also suppressed by the miR-15 family, which, specifically inhibited, has shown to promote myocyte proliferation after myocardial infarction [68]. On the contrary, miR-199 and miR-590 have been found to promote the re-entry of cardiomyocytes in the cell cycle. An interesting therapeutical strategy, supported by preclinical mice studies, would be to inject these molecules into the border zones of infarcted hearts. Positive results and stimulation of cardiomyocytes proliferation have been demonstrated [69,70].

Several miRNAs also regulate the signals of insulin-growth factor 1 (IGF-1) in skeletal muscle, contributing to muscle development or atrophy [71]. Several other studies have noted their regenerative role by observing common cardiovascular pathologies and the subsequent structural remodeling [72]. Long non-coding RNAs are implied in hypertension-related vascular remodeling, post-ischemic recovery, and myocardial hypertrophy [72].

Specific miRNAs are also differentially expressed in bicuspid aortic valve (BAV), the most common congenital heart disease. Aortic valve endothelial cells on the ventricular side are frequently exposed to high shear forces while on the aortic side turbulent blood flow and high levels of antioxidant enzymes are present [73]. Conversely, on the ventricular side, factors inhibiting calcification are more abundant [73,74].

MiR-133 can be used for guiding the therapeutic management of aortic stenosis, due to its potential role in predicting left ventricular hypertrophy [75,76,77]. Sabatino et al. performed a bioinformatic analysis to identify the most commonly regulated miRNAs in normal and stenotic bicuspid aortic valves and compared results with normal and stenotic tricuspid valves for calcium metabolism, blood coagulation, phosphate metabolism, and inflammatory pathways [13]. The authors’ noted that the levels were differentially expressed in bicuspid versus tricuspid aortic valves and were also correlated with the degree of stenosis, as previously discussed. The investigators claim it will be used as a biomarker as it reflects the degree of myocardial fibrosis [13].

The key factor involved in calcium metabolism and inflammatory pathways was found to be pidermal growth factor receptor (EGFR) [78]. Several miRNAs, also associated with calcification, were associated with stenotic tricuspid aortic valves (TAVs) and BAVs, namely miR-100, -130a, -181a/181b, -199a-5p, -199a-3p, and -214 which have been investigated by other authors to display higher expression levels in VECs of the fibrosa on the aortic side, compared to the ventricular side [79].

miR-181 is another important miRNA. Aortic valve endothelial cells were associated with its increased expression but decreased levels of targets, including SIRT1 and GATA6 that negatively affect vascular SMCs elastin production [80]. Several other studies confirmed that its inhibition increases the expression of elastin and collagen while its stimulation, through direct administration, inhibits atherosclerotic lesion formation [81].

## 4. Novel Therapeutical Strategies: miRNAs Targeting to Suppress or Activate Them

### 4.1. Results from In-Vivo and In-Vitro Testing for Aortic Valvular Stenosis

Regulating miRNAs expression is an attractive therapeutic challenge. Valvular calcification is currently not a direct target for pharmacological action. Endothelin receptor antagonists have emerged as possible molecules of interest in preclinical studies [81,82]. Chronic kidney disease-induced valvular calcification was demonstrated to be slowed by administration of endothelin type A (ETA) receptor atransentan (10 mg/kg/day) which reduced SMC differentiation, calcification, and stiffness [81].

Concerning statin treatment, several studies reported increased rates of calcification [83]. Possible strategies for miRNAs overexpression include miRNA mimics, miRNA expression vectors, and small molecules [52,84]. Instead, negatively regulating mi-RNAs seems to encompass different strategies. Antisense oligonucleotides including locked nucleic acid (LNA)-modified anti-miR, or miRNA sponge vectors can be used to specifically bind to miRNAs [52]. Toshima et al. demonstrated miR-34a as a potential therapeutic target. Its inhibition in human aortic tissue exhibiting either calcific aortic valve stenosis (CAVS) or aortic regurgitation (AR) attenuated calcification signals in porcine aortic valve interstitial cells (AVICs) compared with miR-control [85]. After performing RNA pull-down assays, miR-34a was demonstrated to directly target Notch1 by binding to Notch1 mRNA 3′ untranslated region [85]. Additionally, miR-34a inhibitor suppressed calcium deposition of aortic valves and cardiac hypertrophy, both mechanisms involved in decreased Runx2 and increased Notch1 expressions [85].

**Figure 2:**
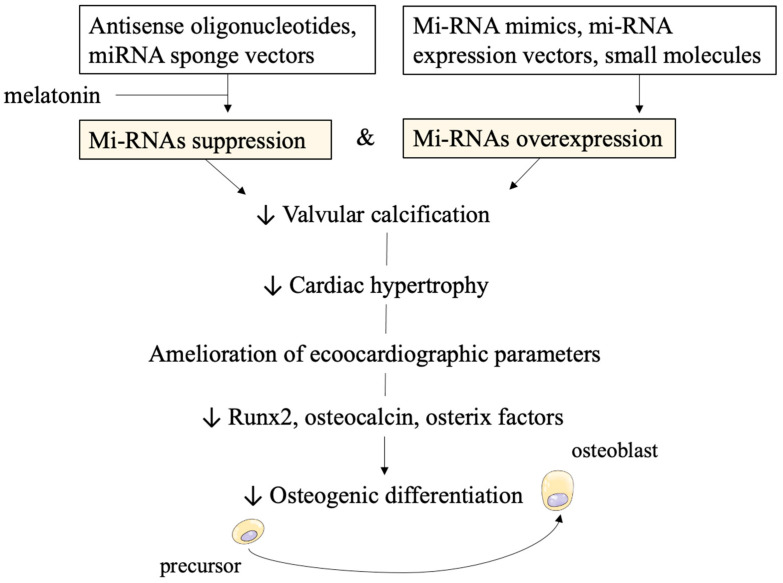
Possible therapeutic strategies and in vitro/in vivo effects of miRNAs targeting. Technological approaches of suppressing and overexpressing miRNAs are displayed at the top of the figure. The subsequent reduction in calcification clinical parameters and molecular pathways is achieved.

Another possible strategy recently proposed, involves melatonin administration. In vitro studies confirmed melatonin reduces the level of CircRIC3, a circular RNA with procalcific effects. It acts as a miR-204-5p sponge to stimulate and increase expression levels of the procalcification gene dipeptidyl peptidase-4 (DPP4). A preclinical in vivo study [86] involving high cholesterol diet (HCD)-treated ApoE^−/−^ mice with aortic valve calcification demonstrated that the intragastric administration of melatonin for 24 weeks improved aortic valvular parameters. It reduced thickness and calcium deposition in the leaflets and ameliorated echocardiographic markers, namely transvalvular peak jet velocity and aortic valve area [86]. At the molecular level, it decreased Runx2, osteocalcin, and osterix factors which are involved in osteogenic differentiation, as we discussed above (Figure 2).

The authors also demonstrated melatonin caused in vitro suppression of calcification in human valvular interstitial cells (hIVICs) [86].

### 4.2. Technical Concerns on Stability and Efficacy of miRNAs as Therapeutical Targets

The primary concerns in utilizing miRNAs as therapeutic targets, either to positively or to negatively affect them, arise from the need of achieving stability and resistance to degradation enzymes. We named expression vectors, antisense nucleotides (ASOs), small molecules and miR-mimics as novel approaches under current experimentation. Expression vectors are also defined as miRNAs sponges and constitute artificial binding sites for miRNAs to reduce their effect on miRNAs [87,88]. Oligonucleotides also bind miRNAs but are regarded as anti-miR for their sequence complementarity. With this strategy, they relieve miRNA targets from degradation or transcriptional blockage. Small molecules serve as translational regulators instead, but specific targets have not been revealed yet.

A possible strategy to improve stability is the modification with 2-O-methyl (2-OMe) [89]. This can be then further stabilized with sulfur atoms in place of non-bridging oxygen atoms in the phosphate backbone. Serum nucleases, deputed to degradation of mi-RNAs, would find it difficult to cleave phosphorothioate bonds, given that they normally cleave phosphate bonds [89,90]. The adding of a 3′ cholesterol tail is another approach to ameliorate stability and efficacy.

To decrease nuclease degradation, modifications including 2-O-methoxyethyl (2-MOE), 2-fluoro (2-F), and locked nucleic acid (LNA) have also been tested [84]. In particular, 2′-F-modifications yield resistant nucleotides only in combination with phosphorothioate modifications and proved to be the most effective one [91].

### 4.3. Disadvantages in Pharmacokinetics and Proposed Mechanisms for Delivery Vehicles

In vitro studies conducted on oligonucleotides had limited pharmacological effects due to unfavorable kinetic characteristics, notably poor tissue distribution and fast excretion. Thus, appropriate delivery systems have been developed, functioning as carriers for in vivo molecular directing [87].

A good delivery system should achieve the following features: evading the immune system response, avoiding nucleases degradation, directed to target cells, and releasing the content for incorporation into RNA processing machinery [92,93,94,95]. The main combination strategies include polymers, lipids, conjugation, antibodies, nanoparticles, and microbubbles [87]. In particular, nanoparticles can deliver anti-miRNAs and small molecules with a greater degree of multi-functionality [96]. The advantages of nanoparticles include large surface-to-volume ratios, hence, controlling their surface properties is crucial [97]. Surface charges also appear fundamental: macrophage scavenging is increased when the charge increases in number (either positive or negative) [96,98,99]. So, minimizing interactions to non-target sites via, as an instance, steric stabilization, would prevent nanoparticles from directing the molecules to undesired locations, and evading the immune system.

Other emerging technologies to improve kinetic parameters are nanoscale drug delivery systems using liposomes [100]. Lower systemic toxicity has also been proved, especially in achieving high efficacies for anticancer therapies [101,102,103]. Microbubbles, instead, have been used in combination with ultrasound to deliver anti-miRNAs after ischemia towards myocardiocytes of mice models. Molecular structure of microbubbles includes mixing of 1,2-distearoyl-sn-glycero-3-phosphocholine, 1,2-stearoyl-3-trimethylammonnium-propane and polyoxyethylene-40-stearate in H_2_O, glycerol, and propylene glycol, in the presence of perfluorobutane gas [87,104,105,106,107].

Local miRNA delivery results have been published and discussed in an Israeli study for metastatic breast cancer prevention by miR-96 and miR-182 treatment [108]. In vivo, local targeting was achieved by coating breast tumor cells with adhesive hydrogel scaffold covered in nanoparticles carrying the miRNAs of interest [108]. Nanoparticle stability achieved with hydrogel was also described for drug delivery in several other studies [109,110,111].

## 5. Conclusions: Future Directions and Clinical Relevance of Preclinical Studies

An Italian group discussed the potential use of miRNAs for mitral valve diseases. In fact, miRNAs appear as predictive diagnostic and prognostic biomarkers but, most promisingly, as potential targets for personalized treatments [112].

**Figure 3:**
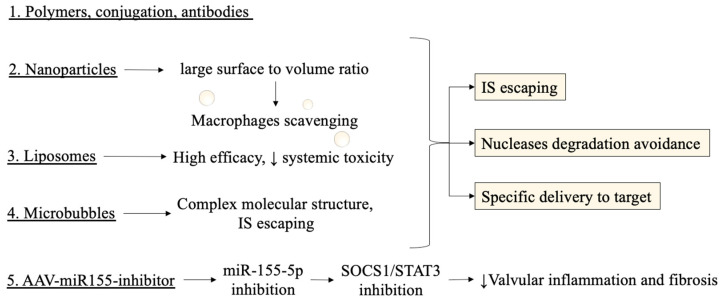
Novel methods and delivery systems for targeting miRNAs are depicted with their respective advantages. IS escaping, nucleases degradation, and specific delivery are features to be achieved by an optimal delivery system. The pathophysiological conclusion from a study regarding AAV-miR155-inhibitor, an adenoviral-based miRNA inhibitor, discussed below in the text, is also reported. IS: immune system, AAV: adeno-associated virus, SOCS1: suppressor of cytokine signaling 1, STAT3: signal transducer and activator of transcription 3.

With regards to the structural and molecular changes in extracellular matrix composition, the miRNA investigations may be relevant in knowing the progression of altered ECM during aging. This process leads to calcification or myxomatous degeneration of cardiac valves and a preventive diagnosis based on miRNA might establish a pharmacological therapeutic target or a basis to improve existing prosthetic devices and treatment options [113,114,115]. An important role could be played by miRna in tissue remodelling secondary to pressure stressors [116,117]. Another impetus from the miRNA study can be given to shed light on the early calcification of cryopreserved tissues [118] (Figure 3).

At present, no clinical data on therapeutic approaches targeting miRNAs are available and hypotheses formulation for future miRNAs practical use remains difficult.

A preclinical study [119] investigated the use of recombinant adeno-associated virus (AAV-miR155-inhibitor) to inhibit the expression of miR-155-5p for valvular damage caused by rheumatic heart disease (RHD) in rat models. The different method, comprising an adenoviral delivery, enabled promising results to be demonstrated. The authors demonstrated how the administration of a miR-155-5p inhibitor prevented activation of the SOCS1/STAT3 signal pathway and this resulted in suppression of valvular inflammation. The authors utilized dual-luciferase assays to display targeting by miR-155-5p of S1PR1 and SOCS1 [118].

Additionally, the reduction in valvular inflammation was detected as decreased tissue levels of Il-6 and Il-17 and fibrosis, both in valves and rat serum.

Unfortunately, even though preclinical studies are very promising, further evidence is needed to understand how signaling pathways directly modulate the pathophysiology of degenerative mitral and aortic valve diseases. Furthermore, with regard to degenerative disease of the mitral valve, more in-depth investigations are needed to clarify the possible role of circulating miR-150-5p and define its causal relationship with the different pathophysiological expressions of degenerative mitral valve prolapse. The main objective is to direct both the early diagnosis and the most suitable therapies through the design and application of clinical experimental and observational studies.

New insights into circulating miRNAs may open up new scenarios for treatment and diagnostic screening. About the therapy, the possibility of identifying different possible pharmacological targets able to slow down or even stop the progression of aortic and mitral degenerative diseases is outlined. Instead, regarding the new diagnostic procedures, miRNAs can be useful as specific circulating molecular signatures to be used as a rapid first line and less expensive screening tool for the identification of severe aortic and mitral disease.

We hope that in the coming years, further emphasis, interest, and funding will be allocated for these fascinating molecular approaches.

## Figures and Tables

**Table 1:** The main results from preclinical studies testing miRNAs are tabulated. Stages B and C from Hulanicka et al. [17] respectively stand for asymptomatic and mild/moderate heart failure. Group B and C from Li et al. [18] represent myxomatous mitral valve disease (MMVD) stages B1 and B2 for group B, stages C and D for group C. ACVIM; American College of Veterinary Internal Medicine, mi-R: microRNA.

Study Groups Hulanicka et al. [13], Li et al. [14]	miR-30b, miR-29b	miR-133b, miR-21, miR-126, miR-423, miR-125, miR-208a, miR-208b	cfa-miR-302d, cfa-miR-380, cfa-miR-874, cfa-miR-582, cfa-miR-490, cfa-miR-329b, and cfa-miR-487b	cfa-miR-103, cfa-miR-98, cfa-let-7b, and cfa-let-7c
ACVIM stage B HF [17]	downregulated	normal	not tested	not tested
ACVIM stage C HF [17]	downregulated	downregulated	not tested	not tested
Group B (stage B1, B2) [18]	not tested	not tested	downregulated	upregulated
Group C (stage C, D) [18]	not tested	not tested	downregulated	upregulated

**Table 2:** Concordant results from Bulent et al. [19] and Songia et al. [53]. Patients affected by MTCR were found to have lower levels of miRNA 150-5p with respect to controls while miR-223-3p in MVP and MTCR groups was found to be lower than controls. (Slashes indicate that some results of miRNA testing on MCTR were not available). MVP: mitral valve prolapse, MCTR: mitral chordae tendineae rupture.

Cardiovascular Diseases	140-3p	150-5p	210-3p	451a	487a-3p	223-3p	323a-3p	361-5p	340-5p
**MVP versus controls**	↑	↑	↑	↑	↑	↓	↓	↓	↓
**MCTR versus controls**	/	↓	/	/	/	↓	/	/	/

## Data Availability

Not applicable.

## Abbreviations

AAV	adeno-associated virus
ACP5	acid phosphatase 5
ACVIM	American College of Veterinary Internal Medicine
ADAMTS-7	a disintegrin and metalloproteinase with thrombospondin motifs-7
AGEs	advanced glycation end products
Ap	alkaline phosphatase
APF	autophagy promoting factor
APPAT	atherosclerotic plaque pathogenesis associated transcript
AR	aortic regurgitation
ASD	atrial septal defects
ASOs	antisense nucleotides
ATF4	activator transcription factor 4
AUROC	Area Under the Receiver Operating Characteristics
AVICS	aortic valve interstitial cells
BAV	bicuspid aortic valve
BMPs	bone morphogenetic proteins
BMP-2	Bone morphogenetic protein 2
CAIF	cardiac autophagy inhibitory factor
CARL	cardiac apoptosis-related lncRNA
CAVS	calcific aortic valve stenosis
Chast	cardiac hypertrophy-associated transcript
CHD	Congenital heart diseases
CHRF	cardiac hypertrophy-related factor;
CircRIC3	circular RNA
CoA	coarctation of the aorta
CPLL	combinatorial peptide ligand library
DPP4	dipeptidyl peptidase-4
EGFR	Epidermal growth factor receptor
ESI-MS/MS	electrospray ionization mass spectrometry
ETA	endothelin type A
FGF	Fibroblast growth factor
GAS5	growth block specificity 5
GATA6	GATA Binding Protein 6
Giver	growth factor-and proinflammatory cytokine-induced vascular cell-expressed lncRNA
Gpx1	Glutathione Peroxidase 1
HCD	high cholesterol diet
HDAC4	histone deacetylase 4
HIF1A-AS1	HIF1 alpha-antisense RNA1
HOTAIR	HOX antisense intergenic RNA
HOXA2	Homeobox A2
hVICs	human valvular interstitial cells
IGF-1	insulin-growth factor 1
IS	immune system
iTRAQ	Isobaric Tags for Relative and Absolute Quantitation
JAK-STAT	Janus kinase-signal transducer and activator of transcription
lincRNA-p21	long intergenic noncoding RNA-p21
LNA	locked nucleic acid MEG3: maternally expressed gene 3
MALAT1	metastasis-associated lung adenocarcinoma transcript 1
Mhrt	myosin heavy chain associated RNA transcripts
MIAT	myocardial infarction–associated transcript
miRNA	microRNA
MITF	Microphthalmia-associated transcription factor
MMPs	Matrix metalloproteinase
MMVD	myxomatous mitral valve disease
MR	mitral regurgitation
MTCR	mitral chordae tendineae rupture
MV	mitral valve
MVP	mitral valve prolapse
NADPH	nicotinamide adenine dinucleotide phosphate
Neat1	nuclear-enriched abundant transcript 1
NRAS	neuroblastoma RAS
OC	osteocalcin
OPG/RANK/RANKL	osteoprotegerin/receptor activator of nuclear factor-kB and its ligand
OPN	Osteopontin
OSX	osterix factor
Ph	phosphorus
PIK3R2	phosphoinositide-3-kinase regulatory subunit 2
Plekhm1	pleckstrin homology domain-containing protein family M member 1
PPP3CA	Protein Phosphatase 3 Catalytic Subunit Alpha
PPP3R1	Protein Phosphatase 3 Regulatory Subunit B, Alpha
Prdx1	Peroxiredoxin 1
PTGS 2	Prostaglandin-Endoperoxide Synthase 2
PVA	pulmonary valve atresia
RHD	rheumatic heart disease
ROS	reactive oxygen species
RT-qPCR	Real-time quantitative PCR
Runx2	RUNX Family Transcription *Factor* 2
SATB2	Special AT-rich sequence-binding protein 2
siRNAs	short interfering RNAs
SIRT1	Sirtuin 1
SM	smooth muscle
SM22α	smooth muscle 22 alpha
SOCS1	suppressor of cytokine signaling 1
SOD1	superoxide dismutase 1
SOD2	superoxide dismutase 2
SRF	serum response factor
STAT3	signal transducer and activator of transcription 3
S1PR1	Sphingosine-1-Phosphate Receptor 1
TA	tricuspid atresia
TAV	tricuspid aortic valve
TGFB1	tumor growth factor-beta 1
TGFBR2	transforming growth factor beta receptor 2
TIMP-2	Tissue inhibitor of metalloproteinases 2
TNF-α	Tumor necrosis factor-alpha
TOF	tetralogy of Fallot
TRPV1	transient receptor potential vanilloid type 1
TUG1	taurine upregulated 1
UCA1	urothelial carcinoma-associated
VC	valvular calcification
VEGFA	vascular endothelial growth factor alpha
VSD	ventricular septal defects
VSMCs	vascular smooth muscle cells
XIST	X-inactive specific transcript
α-SMA	alpha-smooth muscle actin
2D	bi-dimensional
2-OMe	2-O-methyl
3D	three-dimensional
